# H19 lncRNA regulates keratinocyte differentiation by targeting miR-130b-3p

**DOI:** 10.1038/cddis.2017.516

**Published:** 2017-11-30

**Authors:** Chun-xiao Li, Hua-guo Li, Lin-ting Huang, Yu-wei Kong, Fu-ying Chen, Jian-yin Liang, Hong Yu, Zhi-rong Yao

**Affiliations:** 1 Department of Dermatology, Xinhua Hospital, Shanghai Jiaotong University School of Medicine, Shanghai, China

## Abstract

Aberrant differentiation of keratinocytes has been demonstrated to be associated with a number of skin diseases. A growing number of studies have showed that long noncoding RNAs (lncRNAs) have an important part in gene regulation, however, the role of lncRNAs in keratinocyte differentiation remains to be largely unknown. In the present study, we demonstrated that lncRNA-H19 act as an endogenous 'sponge', which binds directly to miR-130b-3p and therefore inhibits its activity on Dsg1. MiR-130b-3p was illustrated to inhibit keratinocyte differentiation by targeting Dsg1. H19 regulates Dsg1 expression and the consequent keratinocyte differentiation through miR-130b-3p. Our study casts light on a novel regulatory model of keratinocyte differentiation, which may provide new therapeutic targets of skin diseases.

The human epidermis, which is a stratified epithelial tissue built of keratinocytes, continuously renews itself approximately every 4 week by a process of keratinocyte migration, proliferation and differentiation. Transient amplifying cells, which are generated from basal epidermal stem cells, move outward from the basal membrane, migrate through the epidermis and undergo terminal differentiation.^1, 2^ A balance between the progenitor compartment and terminally differentiated layers is of great importance for the maintenance of the functional epidermis. Aberrant differentiation of keratinocytes has been demonstrated to be associated with skin diseases, such as psoriasis and atopic dermatitis.^3, 4^

MicroRNAs (miRNAs) are a class of small noncoding RNAs (single-stranded RNAs consisting of 19–22 nucleotides) that act as negative regulators of gene expression at the post-translational level by promoting mRNA degradation or inhibiting mRNA translation.^5^ Accumulating evidence has demonstrated the vital roles that miRNAs have in the regulation of diverse developmental and cellular processes.^4, 5, 6^ They also participate in the regulation of keratinocyte proliferation and differentiation.^4, 7, 8, 9^ Given the important role of miRNAs in the skin disease,^10^ it is of great importance to identify miRNAs that are involved in the regulation of keratinocyte differentiation and to illustrate the underlying signal transduction pathways in the differentiation cascades.

miRNAs are known to exert their effects through targeting protein-coding genes. Although it has been reported that the expression levels of a variety of miRNAs can be altered during the keratinocyte differentiation process,^11^ the target genes of miRNAs remain to be investigated. A number of studies have showed that Desmoglein 1 (Dsg1) had a significant impact on keratinocyte differentiation.^12, 13, 14^ DSG1 promotes keratinocyte differentiation by attenuating MAPK/ERK signaling.^13, 14^ However, it is still unclear whether Dsg1 is a target of miRNAs in the keratinocyte differentiation process.

Long noncoding RNAs (lncRNAs) are a set of RNAs, which are longer than 200 nucleotides in length, but have limited protein-coding potential. lncRNAs participate in a diverse array of cellular processes, and regulate genes expression at the epigenetic, transcriptional and post-transcriptional levels.^15, 16, 17^ The research on lncRNAs’ function in skin diseases has just started.^18, 19^ However, the role of lncRNAs in keratinocyte differentiation remains to be explored.

In this study, we demonstrated that Dsg1 is a target of miR-130b-3p and miR-130b-3p inhibits keratinocyte differentiation through targeting Dsg1. Moreover, our data further illustrate that the lncRNA-H19 may act as an endogenous 'sponge', which binds directly to miR-130b-3p and therefore inhibits its activity. H19 regulates Dsg1 expression and consequently regulates keratinocyte differentiation through miR-130b-3p. Our study suggests a novel keratinocyte differentiation regulatory model in which H19 regulates keratinocyte differentiation via mediating the miR-130b-3p/Dsg1 pathway.

## Results

### MiR-130b-3p participates in the regulation of Dsg1 and inhibits keratinocyte differentiation

**Figure 1:**
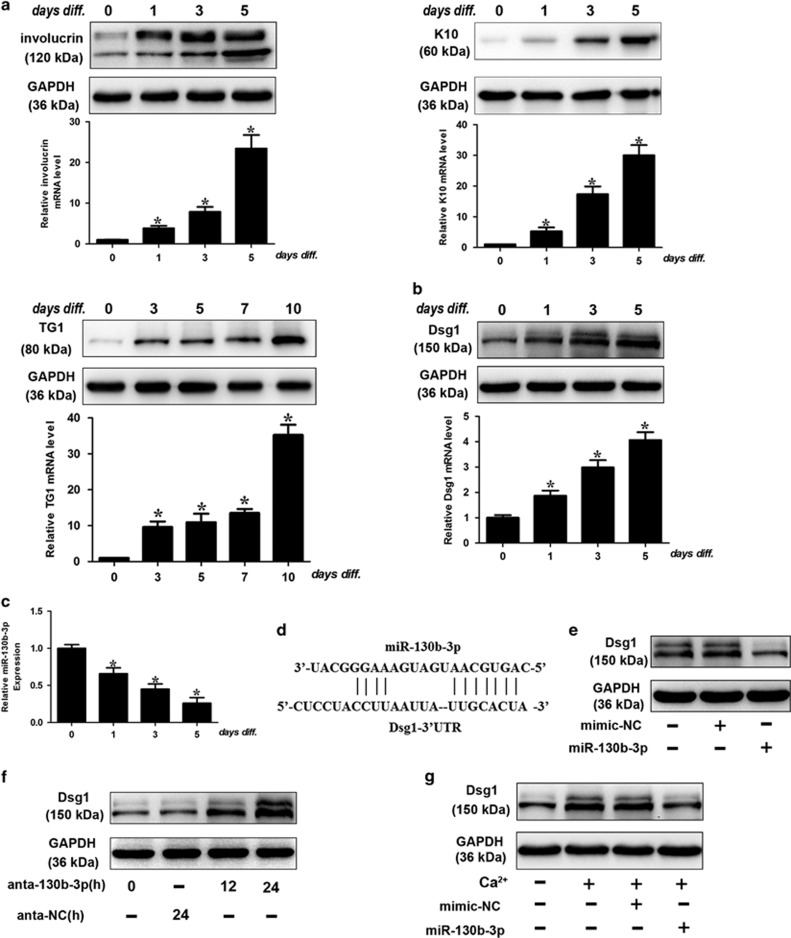
miR-130b-3p participates in the regulation of Dsg1 expression. (**a**) Primary human keratinocytes were isolated as described in Materials and Methods and plated on dishes. Before they reached confluence, cells were induced to differentiate by adding 1.8 mM CaCl_2_ to the culture medium. Cells were collected at the indicated time points to perform western blot analysis. Differentiation was evaluated by western blot for involucrin, K10 and TG1 (**a**). Dsg1 protein (**b**) upregulation during differentiation is also shown; GAPDH is used as loading control. The histograms show the quantitative mRNA level of involucrin, K10, TG1 and Dsg1 (fold over control; black bars) as mean±S.D. from three independent experiments. (**c**) Keratinocytes were exposed to calcium stimulation and miR-130b-3p levels were analyzed by qRT-PCR. Data are shown as mean±S.D. from three independent experiments. (**d**) Putative miR-130b-3p-binding site in the 3’-UTR region of Dsg1. (**e**) miR-130b-3p suppresses the expression of Dsg1. Keratinocytes were transfected with mimic-miR-130b-3p (miR-130b-3p) or mimic negative control (mimic-NC). The levels of Dsg1 were analyzed by immunoblot. *n*=3. (**f**) Knockdown of miR-130b-3p increases the expression levels of Dsg1. Keratinocytes were transfected with miR-130b-3p antagomir (anta-130b-3p) or the antagomir-negative control (anta-NC), the levels of Dsg1 were analyzed by immunoblot; *n*=3. (**g**) Keratinocytes were treated with miR-130b-3p or mimic-NC, and cells were exposed to 1.8 mM CaCl_2_ for 72 h. The levels of Dsg1 were analyzed by immunoblot. *n*=3. *P<0.05 *versus* control in Student’s *t*-test

In the first place, we verified that the primary human keratinocytes could be induced to differentiate by calcium stimulation as demonstrated by quantitative real-time PCR (qRT-PCR) and western blot analysis of early (involucrin, cytokeratin (K)10) and late (transglutaminase (TG)1) markers (Figure 1a). Dsg1 is known to promote keratinocyte differentiation and the expression level of Dsg1 is upregulated during the differentiation process (Figure 1b). Furthermore, the expression level of Dsg1 peaked after Calcium treatment on Day 5 and decreased afterwards (Supplementary Figure 1A). To elucidate the role of Dsg1 in keratinocyte differentiation, we knocked down Dsg1 and found that Dsg1 silencing (Supplementary Figure 1B) retarded keratinocyte differentiation (Supplementary Figure 1C), suggesting that Dsg1 may take effect in the early stage of keratinocyte differentiation.

**Figure 2:**
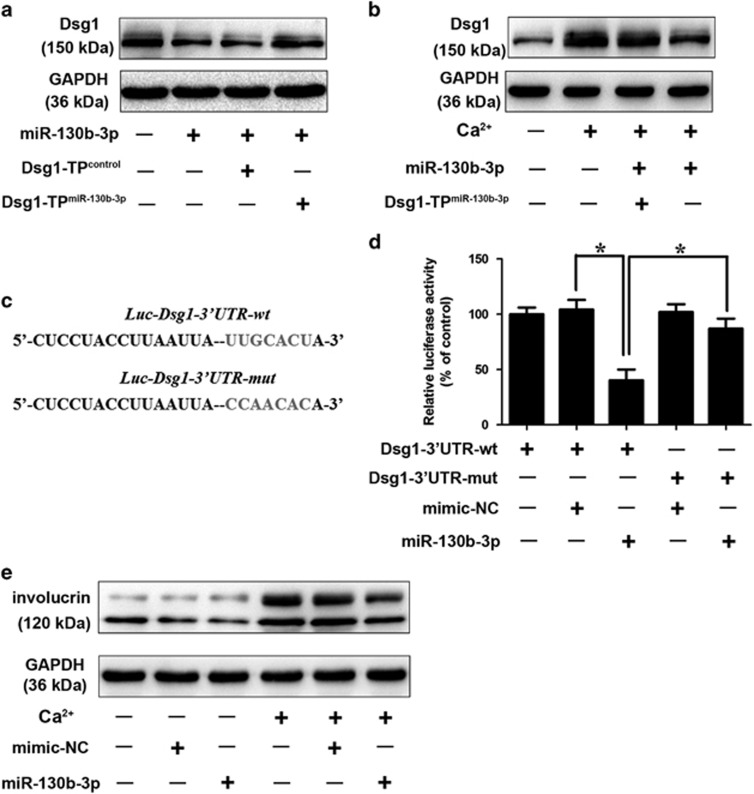
miR-130b-3p participates in the regulation of Dsg1 expression. (**a**) Dsg1 target protector attenuates the reduction of Dsg1 induced by miR-130b-3p. Keratinocytes were transfected with the Dsg1 target protector (Dsg1-TP^miR-130b-3p^) or the control (Dsg1-TP^control^). Dsg1 levels were detected by immunoblot; *n*=3. (**b**) Dsg1 target protector inhibits calcium-induced Dsg1 upregulation. Keratinocytes were transfected with the Dsg1-TP^miR-130b-3p^ or Dsg1-TP^control^, and then exposed to 1.8 mM CaCl_2_ for 72 h. The levels of Dsg1 were analyzed by immunoblot; *n*=3. (**c**) Dsg1 wild-type (WT) 3′-UTR and a mutated 3′-UTR in the miR-130b-3p-binding site are shown. (**d**) miR-130b-3p suppresses Dsg1 translation. HEK293 cells were transfected with miR-130b-3p, mimic-NC, the luciferase constructs of the wild-type Dsg1-3′-UTR (Dsg1-3′-UTR-wt) or a mutated Dsg1-3′-UTR (Dsg1-3′-UTR-mut). The luciferase activity was analyzed. Data are shown as mean±S.D. of three independent experiments. (**e**) Keratinocytes were treated with miR-130b-3p or mimic-NC, and cells were exposed to 1.8mM CaCl_2_ for 72 h. The levels of involucrin were analyzed by immunoblot. *n*=3. *, *P*<0.05 in one-way analysis of variance

miRNAs are a class of short noncoding RNAs that could negatively regulate protein-coding gene expression. To investigate whether Dsg1 could be targeted by miRNAs, the 3′-UTR of Dsg1 was analyzed with TargetScan program and we found a few miRNA-binding sites in 3′-UTR of Dsg1. To explore the miRNAs that are involved in the regulation of Dsg1, we discarded miRNAs that are not significantly altered in response to Calcium stimulation as reported by Hildebrand *et al.*^11^ Among these miRNAs, with Dsg1 upregulation, miRNA-130b-3p was significantly downregulated in differentiating cells (Figure 1c). miRNA-130b-3p obviously reduced the endogenous level of Dsg1 (Figures 1d and e,Supplementary Figure 1D). Antagomir-mediated knockdown of endogenous miR-130b-3p resulted in an increase in Dsg1 expression (Figure 1f,Supplementary Figure 1E). Thus, we further investigated whether Dsg1 is downstream target of miR-130b-3p in regulating keratinocyte differentiation. Under the differentiating conditions, calcium stimulation induced a significant upregulation in Dsg1 expression, whereas ectopic expression of miRNA-130b-3p attenuated the increase in Dsg1 protein level (Figure 1g,Supplementary Figure 1F). A detailed morphologic analysis during keratinocyte differentiation was shown in Supplementary Figure 1G. We used the target protector assay in order to confirm the specificity of the effect of miR-130b-3p on Dsg1. We observed that the target protector of Dsg1 diminished the suppressive effect of miR-130b-3p on Dsg1 (Figure 2a,Supplementary Figure 2A). Target protector of Dsg1 also ameliorated the suppressive effect of miR-130b-3p on Dsg1 in response to calcium stimulation (Figure 2b,Supplementary Figure 2B).

To verify that Dsg1 was indeed a targeting gene of miRNA-130b-3p, we employed the luciferase report assays. A significant reduction in the luciferase activities of wild-type (WT) 3’-UTR of Dsg1 reporter vector was observed after transfected with miRNA-130b-3p mimics. However, the introduction of mutations substantially attenuated the inhibitory effects of miRNA-130b-3p (Figures 2c and d). Our data indicate that miRNA-130b-3p specifically targets Dsg1. Next, we clarified the role of miRNA-130b-3p in the keratinocyte differentiation process. We demonstrated that forced expression of miRNA-130b-3p significantly retarded keratinocyte differentiation as evidenced by ameliorated expression of involucrin (Figure 2e). Rivetti *et al.*^20^ revealed that miR-130b inhibited Np63 expression, which suppressed keratinocyte differentiation.^9^ Yet, miR-130b overexpression in proliferating cells was not sufficient per se to induce senescence.^20^ We found that Calcium treatment induced a significant downregulation of Np63 (Supplementary Figure 2C,D) and resulted in cell senescence (Supplementary Figure 2E). Yet, the anti-proliferative effect of miR-130b-3p was not significant under differentiating conditions.

### H19 directly binds to miRNA-130b-3p and regulates miRNA-130b-3p activity

**Figure 3:**
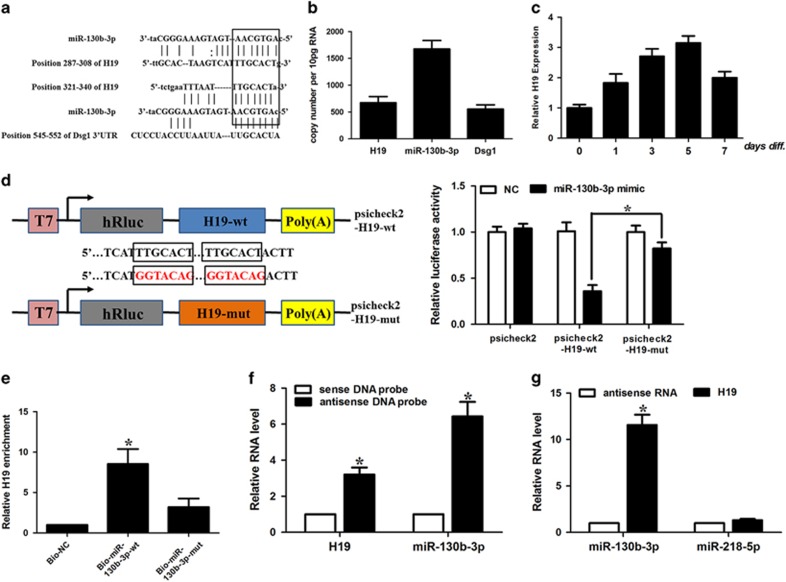
The interaction between H19 and miR-130b-3p. (**a**) H19 RNA contains two sites complementary to miR-130b-3p. (**b**) The copy number of H19 and miR-130b-3p in keratinocytes. (**c**) H19 expression levels on treatment with 1.8 mM CaCl_2_. Keratinocytes were treated with CaCl_2_. H19 expression levels were analyzed by qRT-PCR. Data are shown as mean±S.D. of three independent experiments. *P<0.05 *versus*. control in Student’s *t*-test. (**d**) Schematic representation of psicheck2-based luciferase reporter plasmid containing wild-type H19 (psicheck2-H19-wt) and a mutant reporter construct in which two putative miR-130b-3p-binding sites were mutated (psicheck2-H19-mut), and mutated bases are indicated in red. miR-130b-3p or control mimics were transfected into keratinocytes together with the indicated psicheck2-based luciferase reporter construct. Twenty-four hours after transfection, reporter activity was measured and plotted after normalizing with respect to Renilla luciferase activity. Data shown are means±S.D. (*n*=3; *, *P*<0.05, two-tailed *t*-test). (**e**) miR-130b-3p can bind directly to H19. Keratinocytes were transfected with biotinylated wild-type miR-130b-3p (Bio-miR-130b-3p-wt) or biotinylated mutant miR-130b-3p (Bio-miR-130b-3p-mut). A biotinylated miRNA that is not complementary to H19 was used as a negative control (Bio-NC). Forty-eight hours after transfection, cells were harvested for biotin-based pull-down assay. H19 expression levels were analyzed by real-time PCR. *P<0.05 *versus* Bio-NC. (**f**) Lysates from keratinocytes were incubated with *in vitro* synthesized biotin-labeled sense or antisense DNA probes against H19 for biotin pull-down assay, followed by real-time RT–PCR analysis to examine miR-130b-3p levels. (**g**) Lysates from keratinocytes were incubated with *in* *vitro-*synthesized biotin-labeled H19 and antisense RNA for biotin pull-down assay, followed by real-time RT–PCR analysis to examine miR-130b-3p and miR-218-5p levels

Accumulating evidence provides solid evidence to the hypothesis of competitive endogeneous RNAs (ceRNAs), where lncRNAs could act as endogenous sponge RNA.^21, 22, 23^ We hypothesized that some lncRNA altered during the keratinocyte differentiation process may function as the ceRNA to specifically sponge miRNA-130b-3p and be involved in the keratinocyte differentiation process. We screened the results of two profiling studies during epidermal differentiation process.^24, 25^ Among these lncRNAs, we found that H19 harbored two miRNA-130b-3p-binding sites (Figure 3a) and was consistently upregulated during keratinocyte differentiation process. H19 was shown to be a predominantly cytoplasmic, ~2.3 kb long, capped, spliced and polyadenylated noncoding RNA.^26^ H19 is moderately conserved across species in the putative binding site of miRNA-130b-3p (Supplementary Figure 3). The transcript level of H19 was ~40% of miRNA-130b-3p in keratinocytes and comparable to that of Dsg1 (Figure 3b). Calcium treatment resulted in a time-dependent elevation of H19 (Figure 3c). The expression level of H19 peaked on Day 5 and decreased afterwards. To confirm that H19 binds directly to miRNA-130b-3p, we constructed luciferase constructs containing WT H19 (H19-wt) and a mutated form (H19-mut) (Figure 3d). Luciferase assays revealed that while miRNA-130b-3p could significantly reduce the luciferase activities of reporter containing WT of H19, it had an attenuated effect on the mutant form of H19 (Figure 3d). It suggests that H19 may directly interact with miRNA-130b-3p by the putative binding site.

**Figure 4:**
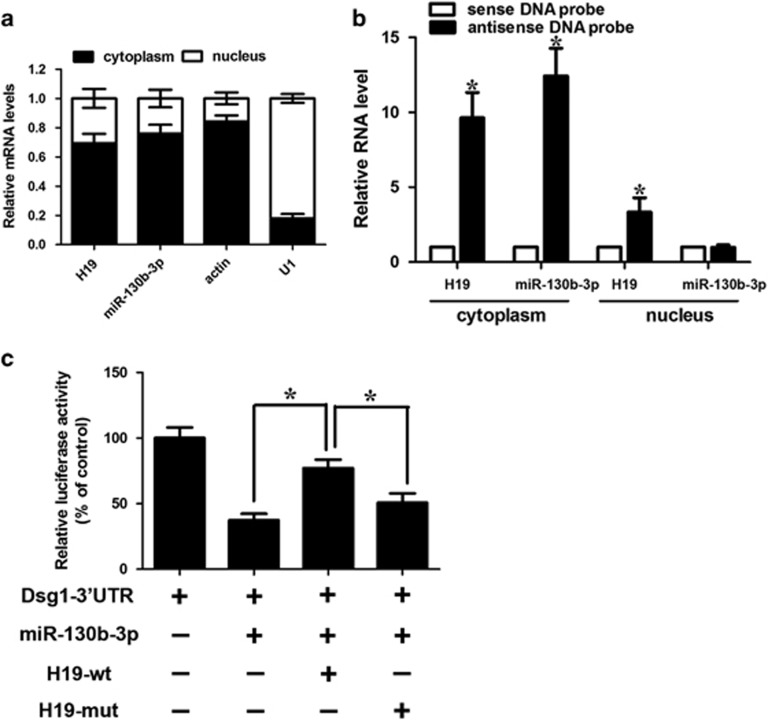
The interaction between H19 and miR-130b-3p. (**a**) Cellular characterization of H19, the levels of nuclear control transcript (U1), cytoplasmic control transcript (Actin) and miR-130b-3p were assessed by qRT-PCR in nuclear and cytoplasmic fractions in keratinocytes. Data are presented as a percentage of U1, Actin, H19 and miR-130b-3p levels and total levels for each were taken to be 100%. Error bars are representative of Standard deviation (S.D., *n*=3). (**b**) Keratinocytes were subjected to cytoplasm or nucleus fractionation before each fraction was incubated with *in vitro*-synthesized biotin-labeled sense or antisense DNA probes of H19 for biotin pull-down assay, followed by real-time RT–PCR analysis to examine miR-130b-3p levels. (**c**) H19 inhibits miR-130b-3p activity. Keratinocytes were infected with adenoviral H19-wt or H19-mut, then transfected with miR-130b-3p and Dsg1-3′-UTR. Luciferase activity was analyzed. Data are shown as mean±S.D. of three independent experiments. *P<0.05 in one-way analysis of variance

Furthermore, the biotin–avidin pull-down assay was used to determine whether miR-130b-3p could specifically pull-down H19. We transfected keratinocytes with biotinylated miR-130b-3p and performed the biotin-based pull-down assay. H19 was pulled down and analyzed with qRT-PCR, but the mutations in the miRNA-130b-3p response elements resulted in the inability of miRNA-130b-3p to pull-down H19 (Figure 3e). It indicates that the recognition of miRNA-130b-3p to H19 is in a sequence-specific manner. We also used *in vitro*-synthesized biotinylated H19 probe and biotinylated antisense DNA probe-enriched endogenous H19 to pull-down miR-130b-3p. MiR-218-5p, which formed no base pairing with H19, was used as a negative control. We showed that H19 specifically pulled down miR-130b-3p (Figures 3f and g), however, H19 was not able to pull-down miR-218-5p (Figure 3g). We tested the subcellular location of H19 and miR-130b-3p. We demonstrated that H19 and miR-130b-3p were both mainly expressed in the cytoplasm (Figure 4a). Antisense DNA probe-enriched endogenous H19 can only pull-down miR-130b-3p from cytosolic but not nuclear fraction (Figure 4b).

To test whether H19 could affect miR-130b-3p activity, we constructed a luciferase reporter containing the 3’-UTR of Dsg1 and transfected it into keratinocytes. Although miR-130b-3p inhibited the luciferase activity of the Luc-Dsg1-3’-UTR, ectopic expression of H19-WT significantly attenuated the suppressive effect of miR-130b-3p, but not the mutant form (Figure 4c). These data indicate that H19 directly binds to miRNA-130b-3p and regulates miRNA-130b-3p activity.

### H19 regulates the keratinocyte differentiation through miR-130b-3p and Dsg1

**Figure 5:**
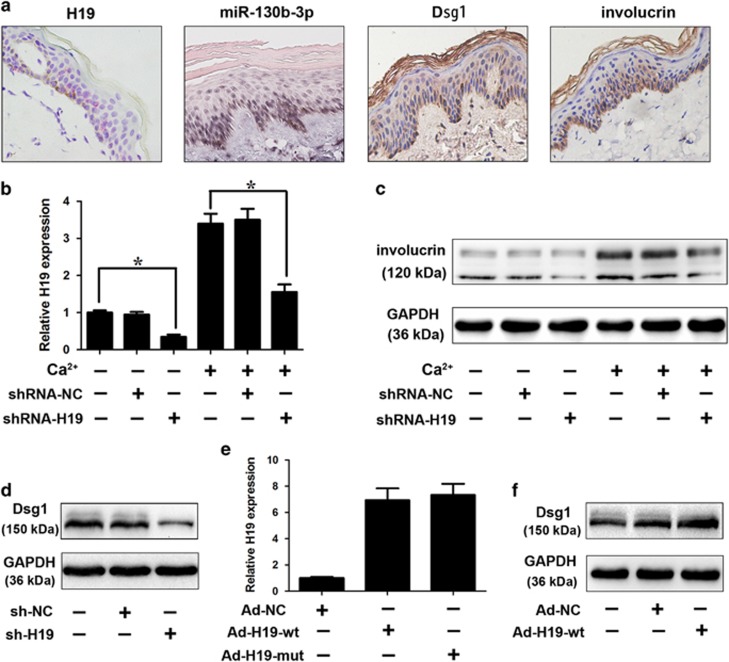
H19 regulates Dsg1 expression and keratinocyte differentiation. (**a**) *In situ* hybridization was performed on human skin using miR-130b-3p or H19-specific probe. Purple color indicates miR—130b-3p expression and red dots indicates H19 expression. Inmmunohistochemical analysis of Dsg1 and involucrin was also performed. (**b**) Keratinocytes were infected with adenoviral H19-shRNA or shRNA-NC. Twenty-four hours after infection cells were treated with 1.8 mM CaCl_2_ for 72 h. H19 levels was analyzed by qRT-PCR. Data are shown as mean±S.D. of three independent experiments. *, *P*<0.05 in one-way analysis of variance. (**c**) Knockdown of H19 inhibits the differentiation of keratinocytes in response to calcium stimulation. Keratinocytes were treated as described in Figure (**b**). Representative immunoblot for diffierentiation marker (involucrin) was shown. (**d**) Knockdown of H19 reduces the expression levels of Dsg1. Keratinocytes were infected with adenoviral H19-shRNA or shRNA-NC. Twenty-four hours after infection, Dsg1 levels were analyzed by immunoblot; *n*=3. (**e**) Enforced expression of H19 increases the expression levels of H19. Keratinocytes were infected with adenoviral H19 (wt and mut). Twenty-four hours after infection, H19 levels were analyzed by real-time PCR. Data are shown as mean±S.D. of three independent experiments. *P<0.05 *versus* control in one-way analysis of variance. (**f**) Enforced expression of H19 induces the increase of Dsg1 levels. Keratinocytes were infected with adenoviral H19. Twenty-four hours after infection, Dsg1 levels were analyzed by immunoblot; *n*=3

**Figure 6:**
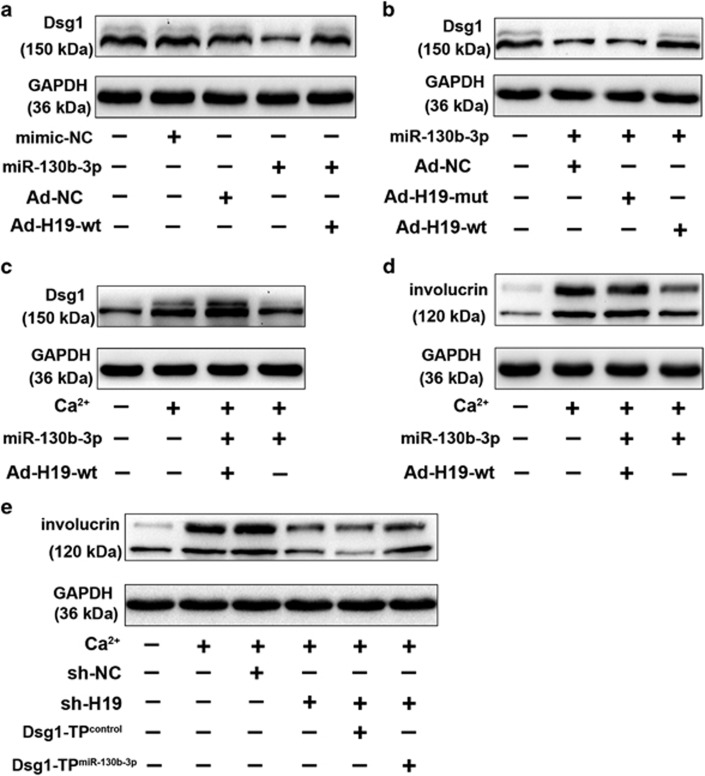
H19 regulates keratinocytes differentiation through miR-130b-3p and Dsg1. (**a**) H19 reduces the inhibitory effect of miR-130b-3p on Dsg1 expression. Keratinocytes were infected with adenoviral H19-wt, and then were transfected with miR-130b-3p. Dsg1 expression levels were analyzed by immunoblot; *n*=3. (**b**) H19-mut has no effect on miR-130b-3p activity. Keratinocytes were infected with adenoviral H19, H19-mut (the binding site of miR-130b-3p in H19 is mutated), then transfected with miR-130b-3p. Dsg1 levels were analyzed by immunoblot. (**c**, **d**) Keratinocytes were infected with adenoviral H19 and then were transfected with miR-130b-3p. Twenty-four hours after infection cells were treated with 1.8 mM CaCl_2_ for 72 h. Dsg1 (**c**) and involucrin (**d**) expression levels were analyzed by immunoblot; *n*=3. (**e**) Dsg1 target protector attenuates the inhibitory effect of H19 knockdown on keratinocyte differentiation induced by calcium stimulation. Keratinocytes were infected with adenoviral H19-shRNA or shRNA-NC, transfected with the target protector (Dsg1-TP ^miR-130b-3p^) or the control (Dsg1-TP ^control^), and then exposed to 1.8mM CaCl_2_ for 72 h. Involucrin levels were analyzed by immunoblot

Consistent with the previous reports ^12, 13, 14^ that Dsg1 was involved in the early-phase keratinocyte differentiation, *in situ* hybridization analysis showed that H19 expression increased during the differentiation process, whereas the expression of miR-130b-3p was downregulated during the keratinocyte differentiation process (Figure 5a,Supplementary Figure 4A). We would like to explore whether H19 regulates keratinocyte differentiation process. Adenovirus-mediated transfection of H19-specific short hairpin RNA (shRNA) significantly downregulated the expression of H19 (Figure 5b). Furthermore, H19 knockdown reduced involucrin levels on calcium stimulation (Figure 5c), suggesting that H19 is involved in the regulation of keratinocyte differentiation. As H19 is able to interact with miR-130b-3p, we examined whether H19 would have any effect on Dsg1. We found that H19 knockdown reduced the expression of Dsg1 (Figure 5d,Supplementary Figure 4B), and ectopic expression of H19 (Figure 5e) contributes to the upregulation of Dsg1 (Figure 5f,Supplementary Figure 4C). H19 attenuated the suppressive effect of miR-130b-3p on Dsg1 expression (Figure 6a,Supplementary Figure 5A). H19-mut had no significant effect on the suppressive effect of miR-130b-3p on Dsg1 expression (Figure 6b,Supplementary Figure 5B). Furthermore, the inhibitory effect of miR-130b-3p on Dsg1 level under differentiating conditions was attenuated with H19 overexpression (Figure 6c,Supplementary Figure 5C). Ectopic expression of H19 ameliorated the suppressive effect of miR-130b-3p on keratinocyte differentiation (Figure 6d). The results suggest that H19 regulates Dsg1 expression through miR-130b-3p. As anticipated, the inhibitory effect of H19 knockdown on keratinocyte differentiation was relieved in the presence of Dsg1 target protector as demonstrated by western blot analysis results (Figure 6e). Furthermore, H19 silencing had no effect on the late phase differentiation (TG1) of keratinocytes (Supplementary Figure 5D).

## Discussion

The epidermis is the first barrier that protects against biological and physical stress induced by the external environment. Homeostasis of skin are maintained by the epidermal stem cell.^1^ Epidermal stem cell gives rise to transient amplifying cell, which periodically move outward in a columnar fashion and terminally differentiate.^2^ Aberrant balance between proliferation and differentiation of keratinocytes have been demonstrated to be associated with skin diseases, such as psoriasis.^3, 4^

Although the roles of miRNAs and miRNAs regulatory network in regulating epidermal stem cell biology have not been fully elucidated, their vital contributions to the process have been demonstrated. Enzymatic complexes Drosha and Dicer are responsible for the maturation of miRNAs through a two-step processing.^26^ Epidermal-conditional depletion of Drosha and Dicer have highlighted the roles of miRNAs in homeostasis of skin.^9, 27^ Increasing number of miRNAs have been shown to take a part in a diversity of cellular processes, in particular, those related to keratinocyte differentiation.^4, 7, 8, 28, 29, 30^ miR-130b-3p have been demonstrated to be functional in lung idiopathic pulmonary fibrosis^31^ and lupus nephritis^32^ via regulating fibroblasts activation. It has also involved in cancer biology.^33, 34^ However, its functional role in keratinocytes and whether it participates in differentiation remain to be elusive. Previous studies have revealed that Dsg1 promotes keratinocyte differentiation via increasing Erbin-SHOC2 interactions, thus attenuating MAPK/ERK signaling.^13, 14^ Our study provides evidence that miR-130b-3p could inhibit keratinocyte differentiation via targeting Dsg1. MiR-130b-3p might be a novel regulator of Dsg1 expression and therefore keratinocyte differentiation.

LncRNAs have been demonstrated to be a novel subclass of ncRNAs. In spite of the fact that lncRNAs may be expressed at lower levels than their counterparts and poorly characterized, they have been demonstrated to exert functions in a variety of physical (development and differentiation)^15, 23, 24^ and pathological processes, including carcinogenesis.^35^ LncRNAs may interact with DNA, RNA or protein and regulate genes at different levels, such as chromatin remodeling, transcription and post-transcriptional processing.^16, 17, 21, 22, 23, 24, 25, 26, 27^ Inspired by the hypothesis of ceRNA,^36^ we explored whether lncRNAs could be involved in the regulation of Dsg1. In the present study, we showed that the expression level of H19 is comparable to that of miR-130b-3p. H19 decreases the activity of miR-130b-3p and consequently increases the expression of miR-130b-3p downstream target Dsg1. Calcium stimulation, which may come from endoplasmic reticulum (ER) Ca^2+^ release in response to barrier perturbation under pathological conditions,^37^ leads to a significant upregulation of H19 and therefore competes with coding mRNA Dsg1 for miR-130b-3p and relieves the inhibitory effect of miR-130b-3p on Dsg1, thereby leading to increased Dsg1 expression, which promotes keratinocyte differentiation. Furthermore, the downregulation of H19 in psoriasis tissues compared with normal tissues was observed in lncRNA profiling studies.^18, 19^

In summary, molecular mechanisms of the regulation of keratinocyte differentiation remains to largely elusive. Our data add evidence to the existence of H19/miR-130b-3p/Dsg1 axis in regulating keratinocyte differentiation.

## Materials and methods

### Keratinocyte culture

We isolated primary human keratinocytes from fresh postoperative skin samples of children as described previously by Hildebrand *et al.*^11^ Primary human keratinocytes were cultured in EpiLife Medium (catalog No. MEPI500CA, Gibco BRL, Grand Island, NY, USA) supplemented with Human Keratinocyte Growth Supplement (catalog No. S0015, GIBCO). Keratinocytes were maintained in a humidified incubator at 37 °C in the presence of 5% CO_2_. Cells were induced to differentiate by adding 1.8 mM CaCl_2_ to the culture medium. All keratinocytes have been passaged for fewer than 6 passages.

### qRT-PCR analysis

Primary human keratinocytes growth and differentiation were carried as described previously.^11^ Stem-loop qRT-PCR analysis of mature miR-130b-3p was performed on an Applied Biosystems ABI Prism 7500 sequence detection system. Total RNA was extracted utilizing Trizol reagent. Following treatment of DNAse I (TaKaRa, Dalian, China), the quality of total RNA was detected at an A260/A280 ratio using quantified by NanoDrop. RNA was reverse transcribed with reverse transcriptase (ReverTra Ace, Osaka, Japan). We quantified RT products with SYBR Green real-time PCR. The results of qRT-PCR of miR-130b-3p were normalized to that of U6 using the 2^−ΔΔCt^ method, where ΔΔCT=((CT_miRNA_−CT_U6_)_Treatment group_−(CT_miRNA_−CT_U6_)_Control group_). The sequences of U6 primers were: 5′-GCTTCGGCAGCACATATACTAA-3′ (forward); 5′-AACGCTTCACGAATTTGCGT-3′ (reverse). The sequences of primers were as followings, H19: 5′-TTCAAAGCCTCCACGACTCT-3′ (forward); 5′-GCTCACACTCACGCACACTC-3′ (reverse). glyceraldehyde-3-phosphate dehydrogenase (GAPDH): 5′-TGTGTCCGTCGTGGATCTGA-3′ (forward); 5′-CCTGCTTCACCACCTTCTTGA-3′ (reverse). The relative levels of mRNA were normalized to the levels of GAPDH using the 2^−ΔΔCt^ method, where ΔΔCT=((CT_mRNA_−CT_GAPDH_)_Treatment group_−(CT_mRNA_−CT_GAPDH_)_Control group_). Samples were run in triplicate.

### Western blot analysis

Western blot analysis was performed as described previously.^14^ The anti-Dsg1 antibody (1:500, Abcam, Cambridge, MA, USA), anti-involucrin antibody (1:300, Abcam), anti-K10 antibody (1:500, Abcam), anti-TG1 (1:200, Santa Cruz Biotechnology CA, USA) and anti-GAPDH antibody (1:2,000, Abcam) were used in this study. The band intensity of western blotting and the normalization were analyzed using the Image J program (National Institutes of Health, Bethesda, MD, USA).

### Adenoviral constructions

Human H19 (Genebank Accession NR_002196.1) was chemically synthesized and verified by sequencing. The constructs were sequence verified. The adenoviruses encoding H19 were constructed using the Adeno-X expression system (Clontech, Otsu, Japan) according to the manufacturer's instructions. The H19-specific shRNA target sequence is shRNA-H19, sense 5′-CTAGGAGAGTTAGCAAAGGTGACATCTCGAGATGTCACCTTTGCTAACTCTCTTTTTG-3′ and antisense: 5′-AATTCAAAAAGAGAGTTAGCAAAGGTGACATCTCGAGATGTCACCTTTGCTAACTCTC-3′. A scramble form targeting GFP was used as a control, shRNA-control: 5′-CTCTGCTCTTAAAGATAATTT-3′. The adenoviruses were generated utilizing the pSilencer adeno 1.0-CMV System (Ambion, Carlsbad, CA, USA) according to the manufacturer's instructions. HEK293 cells were used for adenoviruses amplification. Adenoviral infection of keratinocytes was performed as described previously.^4^

### Luciferase construction and transfection

For luciferase construction, Dsg1-wt and Dsg1-mut 3′-UTRs, H19-wt and H19-mut was subcloned into the psicheck2 vector. Keratinocytes and HEK293 cells were infected with the desired adenoviruses and transfected with the luciferase constructs by Lipofectamine 3000 (Invitrogen, GrandIsland, NY, USA)-mediated gene transfer. Forty-eight hours post transfections, the relative luciferase activity was determined after normalizing to the Renilla luciferase activity.

### Transfection of antagomir and mimic

miR-130b-3p antagomir, antagomir-negative control (antagomir-NC), miR-130b-3p mimic and the mimic negative control (mimic-NC) were purchased from GenePharma Co. Ltd (Shanghai, China). All the bases were 2’-*O-*methy modified, and the 3’-end was conjugated to cholesterol. All the sequence was as the followings: antagomir -miR-130b-3p: 5′-UGCCAACCUUGCAAGCCGAAG-3′ antagomir-NC: 5′-CAGUACUUUUGUGUAGUACAA-3′ miR-130b-3p mimic: 5′-AAGGGCGUUGGAAUCGGCU-3′ miR-130b-3p mutant: 5′-GACCAACUUUGGCCUGAACCUCCU-3′. Mimic control: 5′-CAAGUACUUUGUGUGUAGUACAA-3′. Cells were transfected with the antagomir or mimic at a concentration of 100 nM/ml. The transfection was performed utilizing Lipofectamine 3000 (Invitrogen).

### Target protector preparation and transfection

Target protector sequence is complementary to the binding site of miR-130b-3p in target Dsg1. It was designed to interrupt the direct binding between miRNA and mRNA interaction as described previously.^21^ Dsg1-TP^miR-130b-3p^ sequence is 5′-CTTCCTACCGTGGAACGTGATGGCA-3′. Dsg1-TP^control^ sequence is 5′-CCTCTTACCACACTTTCTTAATTA-3′. They were synthesized by GenePharma Co. Ltd, and transfected into keratinocytes utilizing the Endo-Porter kit (Gene Tools, OR, USA).

### Pull-down assay with biotinylated DNA probe or miRNA

The Pull-down assays with biotinylated DNA probe or miRNA were performed as described previously.^20^

### Statistical analysis

All statistical analyses were performed using SPSS 17.0 (SPSS, Chicago, IL, USA). All data were presented as mean±S.D. from three independent experiments. Student’s *t*-test was used for difference comparison unless otherwise noted. A *P*- value <0.05 was considered to be statistically significant.

Detailed Materials and Methods can be found in the Supplementary Materials.
