# Supplementary material for: H19 lncRNA regulates keratinocyte differentiation by targeting miR-130b-3p
Source: Cell Death Dis. 2017 Nov 30;8(11):e3174–. doi: 10.1038/cddis.2017.516 (PMC5775403; doi:10.1038/cddis.2017.516)
Supplement: Supplementary Methods & Materials [file cddis2017516x1.docx]

**Isolation of epidermal cell fractions from human skin biopsies using collagen IV adherence assays**

Human skin biopsies from healthy children donors were used to isolate epidermal stem, transient amplifying and terminally differentiated cells by collagen IV adherence assays. After elimination of fat residues, the epidermis of human skin biopsies was separated from the dermal compartment by dispase (Roche, 2 U/ml) treatment in PBS for 3 hours at 37° C. Epidermal sheets were trypsinized for 10 min yielding single cell suspensions. 10^5^ cells were collected as a total fraction, and epidermal stem cells and transient amplifying cells were isolated by seeding 10^6^ cells in 4 ml medium on Collagen IV coated plates (BD Biosciences). Rapidly adhering (RA) cells, containing the epidermal stem cells, were isolated by incubating the suspensions 7 min at room temperature on collagen-coated plates. After removal of the supernatants, RA cells were washed 3 times with PBS to remove loosely attached cells and cultured for 3-5 days.

The supernatants, containing the non-adhering (NA) transiently amplifying cells as well as an excess of terminally differentiated keratinocytes, were transferred to fresh Collagen IV coated plates and subcultured for 14 days to allow outgrowth of the transient amplifying cell fraction and to eliminates terminally differentiated keratinocytes, which are unable to proliferate. Note that the term 'non-adhering' / NA, used to designate transient amplifying cells, does not indicate the general inability of these cells do adhere to collagen-coated plates, but only the fact that they do so more slowly than the RA cells and thus are unable to adhere during the initial 7 min incubation period. This is due to lower expression of integrins, which facilitate rapid adherence in this assay.

**Absolute quantification Real time RT-PCR**

We measured the absolute copy number using the standard curve method. The cDNA of H19 was cloned into pcDNA 3.1 vector. The vector was linearized and generated sense RNA transcript using in vitro T7 promoter transcription system (Promega). After digested with RNAse-free DNAse and purification, the transcript was quantified using a spectrophotometer and converted to the number of copies as following formula: Copy number/μl = $\frac{A260\times40\times10-9\times(6.02\times1023)}{(n of A \times329.2) + (n of U \times306.2) + (n of C \times305.2) + (n of G \times345.2)+ 159}$ . The quantified RNA was used as the standard of H19. Synthetic miR-130b-3p RNA was used as the standard of miR-130b-3p. For detection of H19, the standard and total RNA were reverse transcribed with ReverTra Ace (Toyobo) using the reverse primer. The standard of miR-130b-3p were reverse transcribed the same as endogenous miR-130b-3p. The standard cDNA was serially diluted in nuclease-free water. Serial dilutions from 10^6^ to 10^1^ copies were used for standard in a final volume of 20 μl alongside a negative control (RNA) and a non-template control. The SYBR® Premix Ex Taq™ II Kit (Takara) was used for amplification. Quantitative PCR was performed on a CFX96 Real-Time PCR Detection System (Bio-Rad). Absolute quantification determines the actual copy numbers of target genes by relating the Ct value to a standard curve. The data were analyzed by CFX96 software. The final data was expressed as the copy number per 10pg of total RNA.

**Preparations of subcellular fractions**

The cells were washed twice with PBS and the pellets were suspended in 0.2 ml of buffer A (20 mmol l^-1^ HEPES pH 7.5, 10 mmol l^-1^ KCl, 1.5 mmol l^-1^ MgCl2, 1 mmol l^-1^ EGTA, 1 mmol l^-1^ EDTA, 1 mmol l^-1^ DTT, 0.1 mmol l^-1^ PMSF and 250 mmol l^-1^ sucrose) containing a protease inhibitor cocktail. The cells were homogenized by 12 strokes in a Dounce homogenizer. The homogenates were centrifuged twice at 750 g for 5min at 4^。^C to collect nuclei and debris. The supernatants were centrifuged at 10,000 g for 15 min at 4^。^C to collect mitochondria-enriched heavy membrane pellet. The resulting supernatants were centrifuged to yield cytosolic fractions.

**Pull-down assay with biotinylated miRNA**

To evaluate the specificity of the interaction between H19 and miR-130b-3p, we performed pull-down assay as described previously [20]. Briefly, keratinocytes were transfected with synthesized biotinylated miRNA (50 nM) (GenePharma Co. Ltd, Shanghai, China) and analyzed 48 h post transfection. After being washed by PBS, keratinocytes were prepared using in a lysis buffer [20 mM Tris, pH 7.5, 200 mM NaCl, 2.5 mM MgCl2, 0.05% Igepal, 60 U/mL Superase-In (Ambion), 1 mM DTT and a protease inhibitor cocktail on ice for 10 min. The lysates were precleared by centrifugation, and 50μl of the samples were aliquoted for input. The remaining lysates were incubated with M-280 streptavidin magnetic beads (Sigma). To prevent non-specific binding of RNA and protein complexes, the beads were coated with RNase-free BSA and yeast tRNA (both from Sigma). The beads were incubated at 4℃ for 3h, washed twice with ice-cold lysis buffer, three times with the low salt buffer (0.1%SDS, 1%Trition X-100, 2 mM EDTA, 20mM Tris-HCl pH8.0, 150 mM NaCl), and once with the high salt buffer (0.1%SDS, 1%Trition X-100, 2 mM EDTA, 20 mM Tris-HCl pH 8.0, 500 mM NaCl). The bound RNAs were purified using Trizol for the analysis.

**Ago2 IP followed by biotin-labeled probe pulldown assay**

To determine whether H19 interacts with miR-130b-3p, we performed RNA pull-down assay using synthesized biotinlabeled H19 as a probe and then detected Ago 2 from the pellet by western or detected miRNA-130b-3p by quantitative RT-PCR (qRT-PCR).

Briefly, the DNA fragment covering has-miRNA-130a seed region binding site of HOTAIR was PCRamplified using a T7 containing primer and then cloned into pCR8 (Invitrogen). In addition, lncRNA loc285194 was also cloned and used in RNA Pull-Down Assay as a positive control. The resultant plasmid DNA was linearized with restriction enzyme Not I. Biotin-labeled RNAs were in vitro transcribed with the Biotin RNA Labeling Mix (Roche Diagnostics, Indianapolis, IN) and T7 RNA polymerase (Roche), treated with RNase-free DNase I (Roche), and purified with the RNeasy Mini Kit (Qiagen, Inc.,Valencia, CA). Cell nuclear extract (2 ug) was mixed with biotinylated RNA (100 pmol). Washed Streptavidin agarose beads (100 ml) were added to each binding reaction and further incubated at room temperature for 1 h. Beads were washed briefly three times and boiled in SDS buffer, and the retrieved protein was detected by standard western blot technique. The coprecipitated RNAs were detected by reverse transcription PCR. Total RNAs and controls were also assayed to demonstrate that the detected signals were from RNAs specifically binding to Ago2.

**Pull-down assay with biotinylated DNA probe**

The biotinylated DNA probe complementary to H19 RNA was synthesized and dissolved in 500 μl of wash/binding buffer (0.5M NaCl, 20 mM Tris-HCl, pH 7.5, and 1 mM EDTA). The probes were incubated with streptavidin-coated magnetic beads (Sigma) at 25 °C for 2 h to generate probe-coated magnetic beads. Cardiomyocyte lysates were incubated with probe-coated beads, and after washing with the wash/binding buffer, the RNA complexes bound to the beads were eluted and extracted for Northern blot analysis. The following primer sequences were used: H19 pull-down probe, GTTGATGGATGATACTAACTATG; and random pull-down probe, TGATGTCTAGCGCTTGGGCTTTG.

***In situ* hybridization**

In situ hybridization of miR-130b-3p was performed on 10-μm-thick frozen sections of human specimens. Briefly, after incubated in acetylation solution (0.02 M HCl, 1.3% trietanolamin and 0.25% acetic anhydride in diethyl pyrocarbonate-treated water) for 10 min at room temperature (RT), sections were treated with proteinase K (5 μg ml−1) in PBS for 10 min, washed and prehybridized for 6 h. Hybridization with mmu-miR-31 5’-DIG and 3’-DIG-labelled miRCURY LNA detection probe (Exiqon, #39153-15) was performed overnight at 50 °C. Slides were then washed with 5 × SSC buffer followed by incubation in 0.2 × SSC buffer for 1 h at 60 °C. The probe binding was detected by incubating sections with anti-DIG-alkaline phosphatase antibody (Ab) (1:2,000, Roche, #11093274910) for overnight at 4 °C. Sections were visualized by using NBT/BCIP ready-to-use tablets (Roche, #11697471001), according to the manufacturer's instructions. In situ hybridization of H19 was performed with RNAscope® Probe hs-H19 (ACD, #400771) and RNAscope® 2.5 HD Detection Kit (RED) following the manufacturer’s instructions.
