# Supplementary figures and images for: H19 lncRNA regulates keratinocyte differentiation by targeting miR-130b-3p
Source: Cell Death Dis. 2017 Nov 30;8(11):e3174–. doi: 10.1038/cddis.2017.516 (PMC5775403; doi:10.1038/cddis.2017.516)

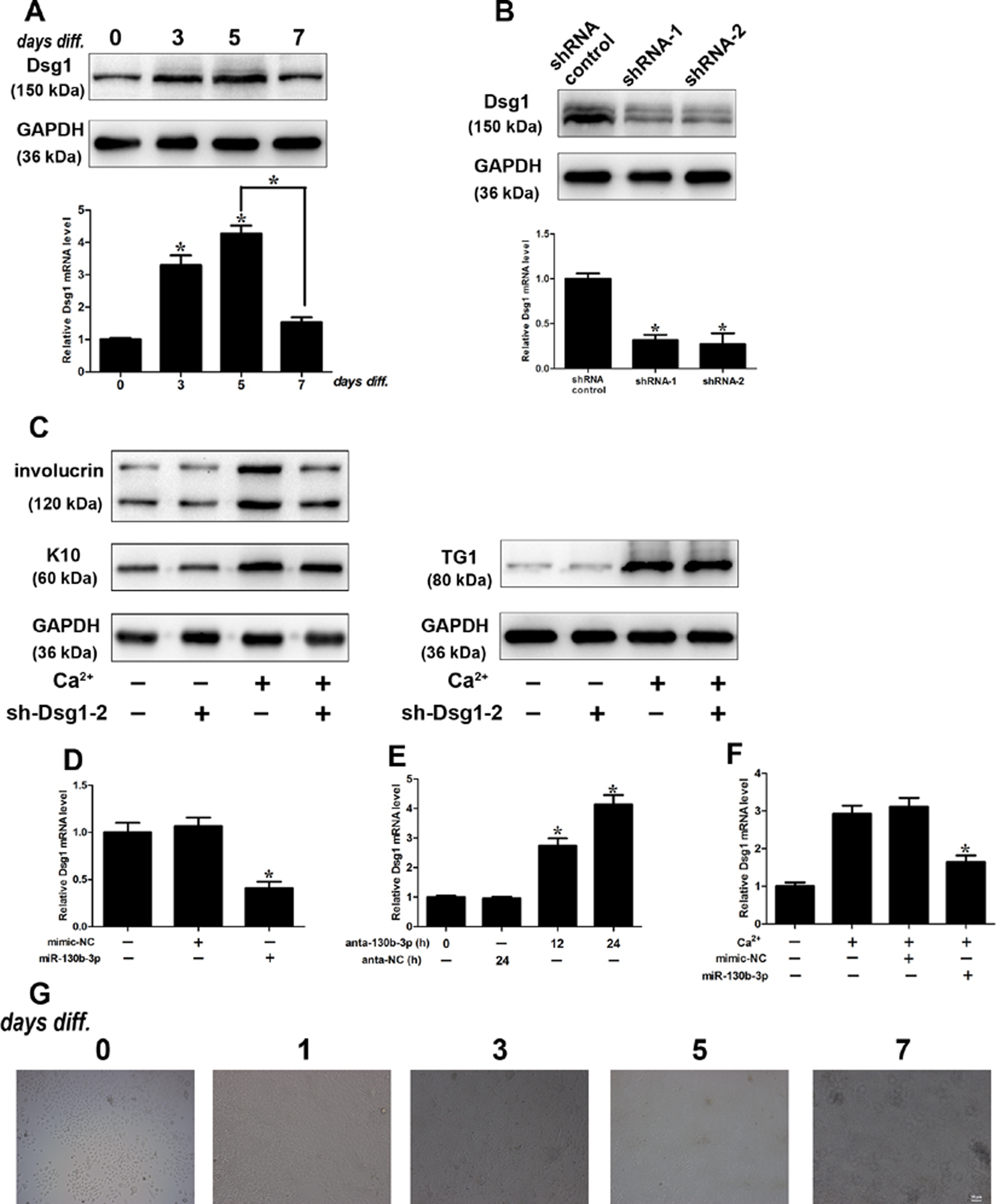

Supplement: Supplementary Figure 1 [file cddis2017516x2.tif]

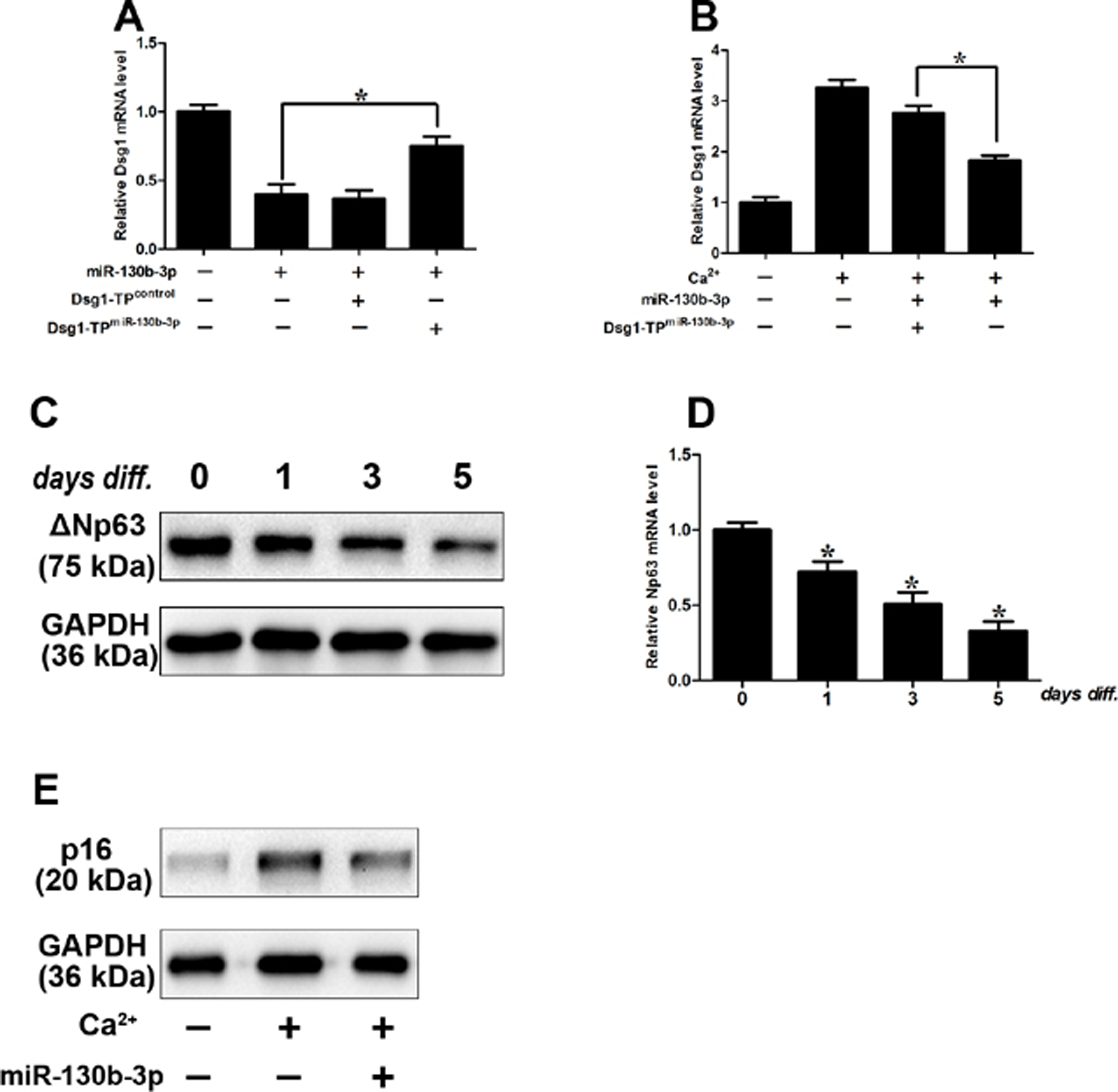

Supplement: Supplementary Figure 2 [file cddis2017516x3.tif]

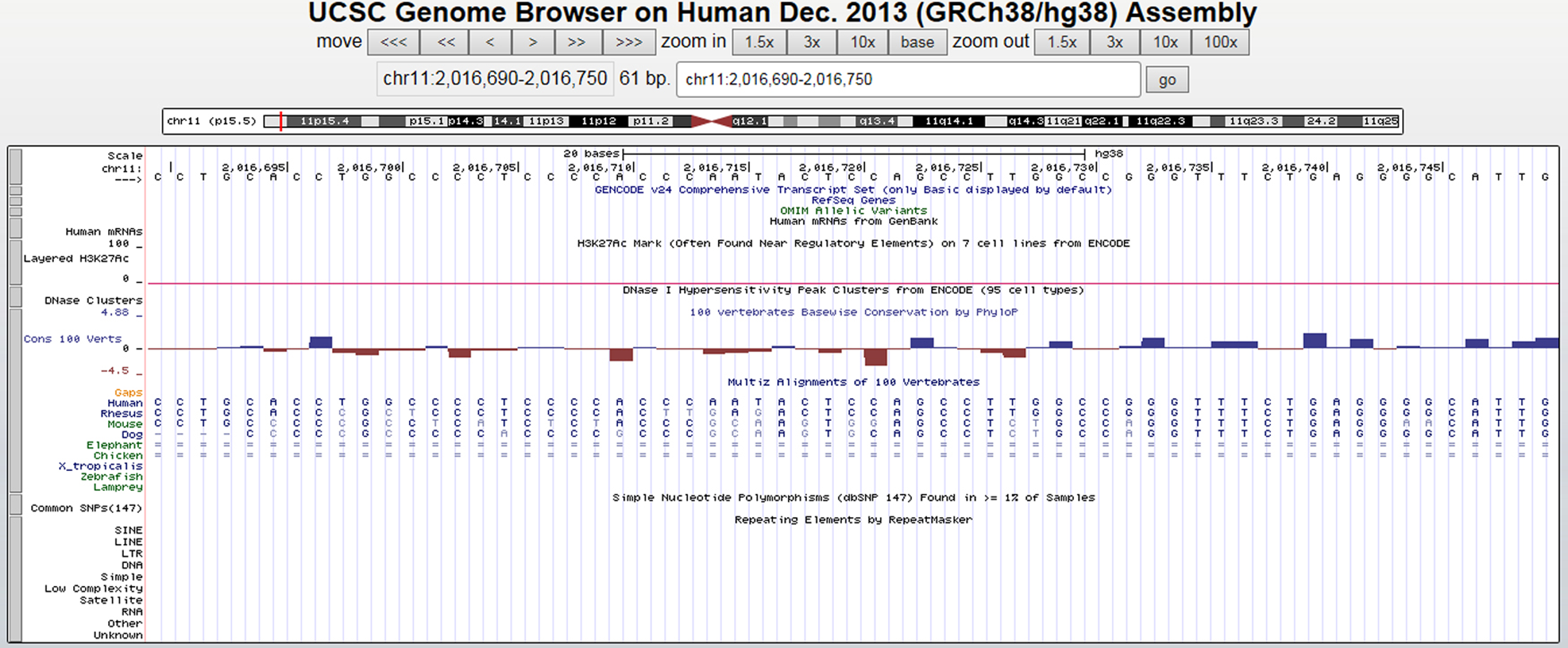

Supplement: Supplementary Figure 3 [file cddis2017516x4.tif]

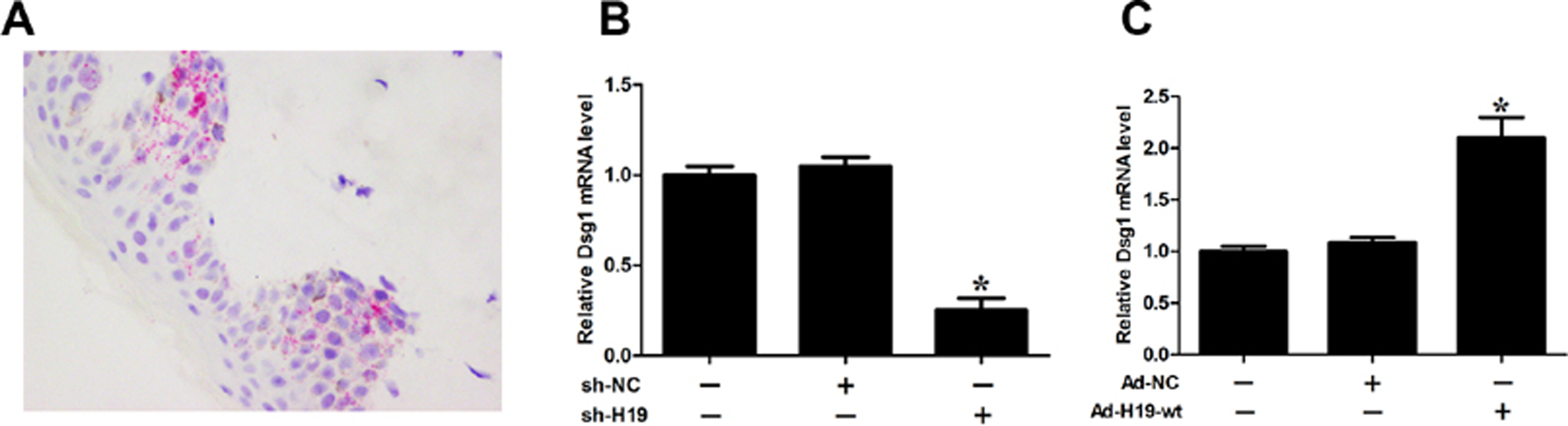

Supplement: Supplementary Figure 4 [file cddis2017516x5.tif]

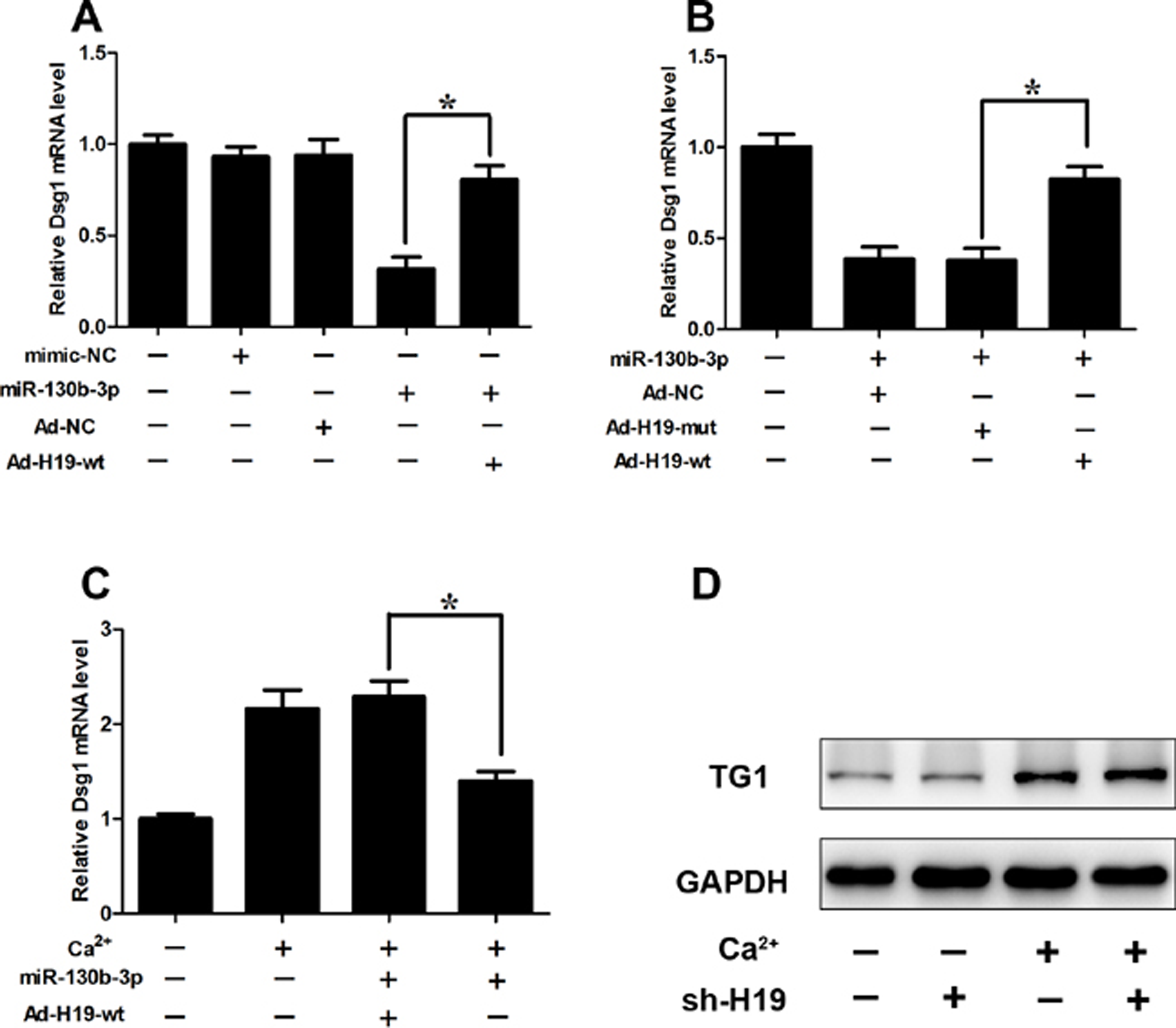

Supplement: Supplementary Figure 5 [file cddis2017516x6.tif]
