# Supplementary material for: H19 lncRNA regulates keratinocyte differentiation by targeting miR-130b-3p
Source: Cell Death Dis. 2017 Nov 30;8(11):e3174–. doi: 10.1038/cddis.2017.516 (PMC5775403; doi:10.1038/cddis.2017.516)
Supplement: Supplementary Figure legends [file cddis2017516x7.docx]

Supplemental Figure 1. **miR-130b-3p participates in the regulation of Dsg1 expression.** (A) Primary human keratinocytes were isolated as described in Materials and Methods and plated on dishes. Before they reached confluence, cells were induced to differentiate by adding 1.8 mM CaCl_2_ to the culture medium. Cells were collected at the indicated time points to perform western blot analysis. Dsg1 was evaluated by western blot analysis. GAPDH is used as loading control. The histograms show the quantitative mRNA level of Dsg1 (fold over control; black bars) as mean±S.D. from three independent experiments. (B) Knockdown of Dsg1 reduces the protein level of Dsg1. Keratinocytes were infected with adenoviral Dsg1-shRNA or shRNA-NC. Twenty-four hours after infection, Dsg1 levels were analysed by immunoblot; n=3. (C) Keratinocytes were treated with adenoviral Dsg1-shRNA or shRNA-NC, and cells were exposed to 1.8 mM CaCl_2_ for 72 hours (involucrin and K10) or 240 hours (TG1). The protein levels were analysed by immunoblot. n=3. *, p<0.05 in one-way analysis of variance. (D) Keratinocytes were transfected with mimic-miR-130b-3p (miR-130b-3p) or mimic negative control (mimic-NC). The levels of Dsg1 were analysed by qRT-PCR. n=3. (E) Keratinocytes were transfected with miR-130b-3p antagomir (anta-130b-3p) or the antagomir-negative control (anta-NC), the levels of Dsg1 were analysed by qRT-PCR; n=3. (F) Keratinocytes were treated with miR-130b-3p or mimic-NC, and cells were exposed to 1.8 mM CaCl_2_ for 72 hours. The levels of Dsg1 were analysed by qRT-PCR. n=3. *, *p*<0.05 versus control in Student’s t-test. (G) Cells were induced to differentiate by adding 1.8 mM CaCl_2_ to the culture medium. A detailed morphologic analysis during keratinocyte differentiation.

**Supplemental Figure 2.** **miR-130b-3p participates in the regulation of Dsg1 expression.** (A) Dsg1 target protector attenuates the reduction of Dsg1 induced by miR-130b-3p. Keratinocytes were transfected with the Dsg1 target protector (Dsg1-TP^miR-130b-3p^) or the control (Dsg1-TP^control^). Dsg1 levels were detected by qRT-PCR; n=3. (B) Dsg1 target protector inhibits calcium-induced Dsg1 upregulation. Keratinocytes were transfected with the Dsg1-TP^miR-130b-3p^ or Dsg1-TP^control^, and then exposed to 1.8 mM CaCl_2_ for 72 hours. The levels of Dsg1 were analysed by qRT-PCR; n=3. (C,D) Primary human keratinocytes were induced to differentiate by adding 1.8 mM CaCl_2_ to the culture medium. Cells were collected at the indicated time points to perform western blot (C) and qRT-PCR (D) analysis of Np63. (E) Keratinocytes were treated with miR-130b-3p or mimic-NC, and cells were exposed to 1.8 mM CaCl_2_ for 72 hours. The levels of p16 were analysed by western blot analysis. n=3. *, *p*<0.05 versus control in Student’s t-test.

**Supplemental Figure 3.** Conservation of H19 in the binding site of miR-130b-3p. This is a snapshot from human genome (2011 assembly) in UCSC Genome Browser.

**Supplemental Figure 4.** (A) In situ hybridization was performed on human skin using H19 specific probe. Red dots indicates H19 expression. (B) Keratinocytes were infected with adenoviral H19-shRNA or shRNA-NC. Twenty-four hours after infection, Dsg1 levels were analysed by qRT-PCR; n=3. (C) Keratinocytes were infected with adenoviral H19. Twenty-four hours after infection, Dsg1 levels were analysed by qRT-PCR; n=3.

**Supplemental Figure 5. H19 regulates keratinocytes differentiation through miR-130b-3p and Dsg1.** (A) H19 reduces the inhibitory effect of miR-130b-3p on Dsg1 expression. Keratinocytes were infected with adenoviral H19-wt, and then were transfected with miR-130b-3p. Dsg1 expression levels were analysed by qRT-PCR; n=3. (B) H19-mut has no effect on miR-130b-3p activity. Keratinocytes were infected with adenoviral H19, H19-mut (the binding site of miR-130b-3p in H19 is mutated), then transfected with miR-130b-3p. Dsg1 levels were analyzed by qRT-PCR. (C) Keratinocytes were infected with adenoviral H19 and then were transfected with miR-130b-3p. 24h after infection cells were treated with 1.8 mM CaCl_2_ for 72 hours. Dsg1 expression levels were analysed by qRT-PCR; n=3. (D) Keratinocytes were treated with adenoviral H19-shRNA, and cells were exposed to 1.8 mM CaCl_2_ for 240 hours. The protein levels were analysed by immunoblot. n=3.
